# A little bit of sex prevents mutation accumulation even in apomictic polyploid plants

**DOI:** 10.1186/s12862-019-1495-z

**Published:** 2019-08-14

**Authors:** Ladislav Hodač, Simone Klatt, Diego Hojsgaard, Timothy F. Sharbel, Elvira Hörandl

**Affiliations:** 1 Department of Systematics, Biodiversity and Evolution of Plants (with Herbarium), University of Goettingen, Goettingen, Germany; 2 Global Institute for Food Security, University of Saskatchewan, Saskatoon, Canada

**Keywords:** Apomixis, Haploid selection, Mutation accumulation, Polyploidy, Plants

## Abstract

**Background:**

In the absence of sex and recombination, genomes are expected to accumulate deleterious mutations via an irreversible process known as Muller’s ratchet, especially in the case of polyploidy. In contrast, no genome-wide mutation accumulation was detected in a transcriptome of facultative apomictic, hexaploid plants of the *Ranunculus auricomus* complex. We hypothesize that mutations cannot accumulate in flowering plants with facultative sexuality because sexual and asexual development concurrently occurs within the same generation. We assume a strong effect of purging selection on reduced gametophytes in the sexual developmental pathway because previously masked recessive deleterious mutations would be exposed to selection.

**Results:**

We test this hypothesis by modeling mutation elimination using apomictic hexaploid plants of the *R. auricomus* complex. To estimate mean recombination rates, the mean number of recombinants per generation was calculated by genotyping three F1 progeny arrays with six microsatellite markers and character incompatibility analyses. We estimated the strength of purging selection in gametophytes by calculating abortion rates of sexual versus apomictic development at the female gametophyte, seed and offspring stage. Accordingly, we applied three selection coefficients by considering effects of purging selection against mutations on (1) male and female gametophytes in the sexual pathway (additive, *s* = 1.000), (2) female gametophytes only (*s* = 0.520), and (3) on adult plants only (sporophytes, *s* = 0.212). We implemented recombination rates into a mathematical model considering the three different selection coefficients, and a genomic mutation rate calculated from genome size of our plants and plant-specific mutation rates. We revealed a mean of 6.05% recombinants per generation. This recombination rate eliminates mutations after 138, 204 or 246 generations, depending on the respective selection coefficients (*s* = 1.000, 0.520, and 0.212).

**Conclusions:**

Our results confirm that the empirically observed frequencies of facultative recombination suffice to prevent accumulation of deleterious mutations via Muller’s ratchet even in a polyploid genome. The efficiency of selection is in flowering plants strongly increased by acting on the haplontic (reduced) gametophyte stage.

**Electronic supplementary material:**

The online version of this article (10.1186/s12862-019-1495-z) contains supplementary material, which is available to authorized users.

## Background

The rate of genomic deleterious mutation accumulation and its effects are fundamental to the evolution of species [1]. The evolution of mutation rate is shaped by the presence or absence of recombination, which is an important consequence of meiosis and sex [2, 3]. Recombining genomes eliminate deleterious mutations in significantly higher rates than is possible in non-recombining genomes [4, 5] because recombination can segregate multiple deleterious mutations into single linkage groups that can be eliminated [6]. Obligate asexual lineages, in contrast, are expected to accumulate recessive deleterious mutations in an irreversible, ratchet-like manner over generations as the least loaded genotypes are lost by random drift (Muller’s ratchet; [7, 8]). In theoretical models, even low recombination rates slow down mutation accumulation [7, 8]. However, empirical studies on genomic mutation accumulation are still rare, especially for polyploid genomes. Polyploidy multiplies the mutation load by c*U* whereby *c* is the ploidy level and *U* is the mutation rate per haploid genome [9]. Polyploid genomes, however, are typical for many asexual eukaryotes and are particularly common in asexually reproducing plants [10]. A novel hypothesis by [11] for flowering plants predicted that even in high polyploids, low rates of facultative sexuality would suffice to counteract mutation accumulation.

**Fig. 1:**
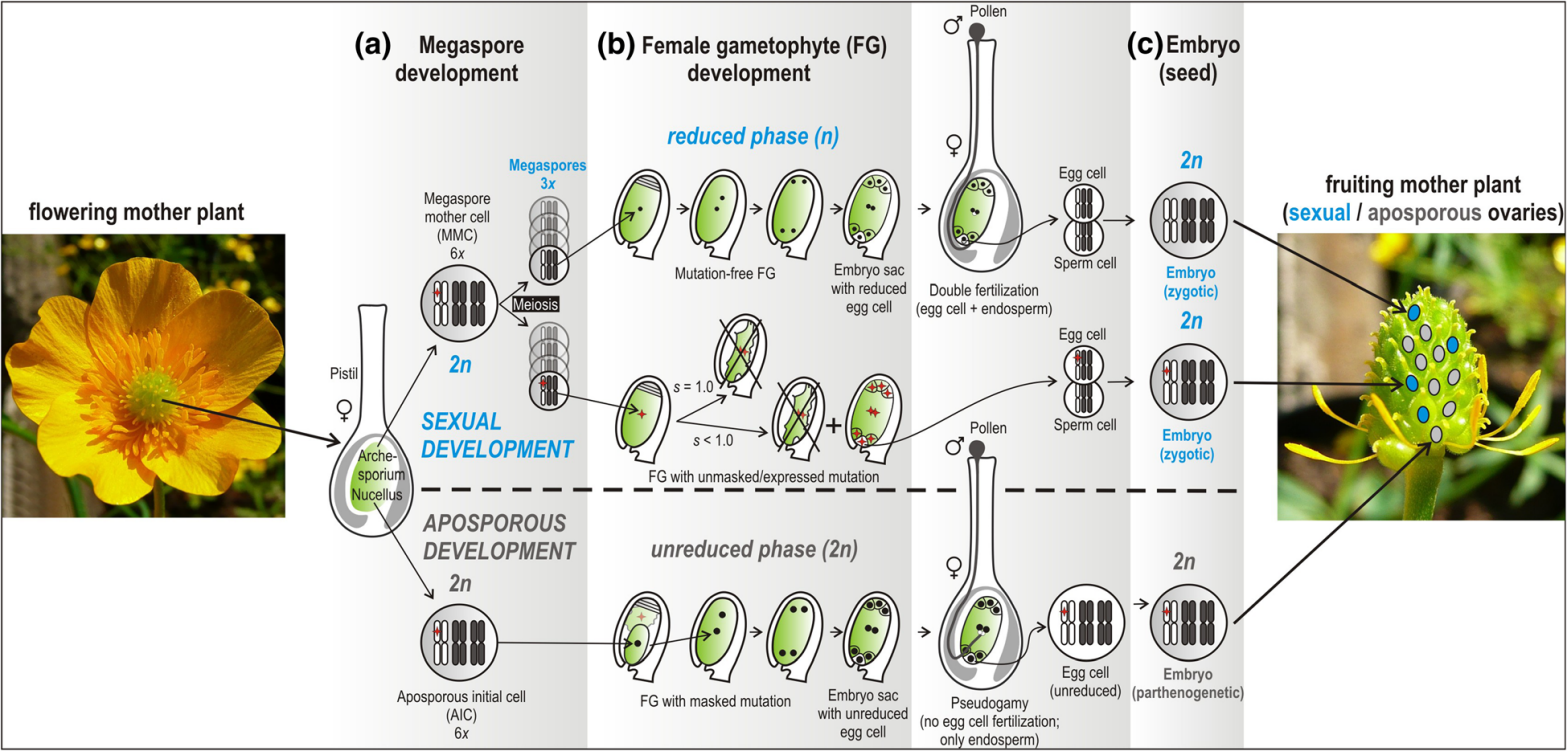
Female gametophyte development in facultative apomictic (aposporous) allohexaploid *Ranunculus auricomus*. The scheme illustrates the parallel development of sexual (upper part) and apomictic (aposporous; lower part) gametophytes and seeds on the same plant (i.e., developmental pathways from megaspore/aposporous initial (**a**) over female gametophyte (**b**) to embryo (**c**). The recessive deleterious mutation is indicated by a red asterisk. In the reduced phase of the sexual development, strong selection (*s* = 1.0) eliminates mutated gametophytes. The mutation is transmitted into embryo and offspring under weaker selection (*s* < 1.0). The aposporous development without reduced phase transmits the mutation into embryo and offspring as the mutation remains masked

This model is based on the consideration of three features specific for flowering plants: First, a sporophyte generation (the familiar green plant) and a gametophyte generation (embryo sac and pollen) alternate during the life cycle, which can increase the efficacy of haploid selection [12]. The female gametophyte remains on the sporophyte, but nevertheless has a separate development. With sexual development, the sporophyte is diplontic (unreduced), while the gametophyte develops from a product of meiosis and hence is haplontic (reduced); in asexual development both sporophyte and gametophyte are diplontic. Diploidy serves to mask the effects of deleterious recessive mutations in heterozygous loci as a non-mutated copy of the respective gene is available on the homologous chromosome [13, 14]. Haploidy eliminates the masking effect and exposes mutations to purging selection, and selection is most efficient in haploids [12, 13]. After a prolonged diploid phase, the return to haploidy leads to the exposure of previously masked deleterious recessive alleles [12, 15]. In plants, gametophytes are multicellular ‘mini’-organisms with significant nuclear gene expression [16, 17], and complex signaling pathways [18]. Consequently, purging selection can strongly act on haploid, or more general, haplontic sexual gametophytes, but to a lesser degree on diploid or diplontic (unreduced) apomictic ones (Fig. 1). Second, plant developmental biology allows for concurrent selection on sexual and asexual development within the same generation. Plants reproducing via apomixis (i.e., clonal reproduction via seed), develop a female gametophyte from an initial cell that has not undergone meiosis. Hence, these egg cells are unreduced, non-recombinant, and develop parthenogenetically into embryos. In parallel (in the same or another ovule of the same plant), meiotically produced megaspores can develop into gametophytes, and the reduced, recombinant egg cells are fertilized; the zygotes develop into embryos (Fig. 1). That means, sexual and apomictic gametophytes/gametes/seeds can be produced by the same mother plant in the same generation [19–22]. Occasional fertilization of egg cells is possible as apomixis affects only female development, while male gametophyte development in the pollen is more or less normal and results mostly in reduced and recombined male gametes [22]. Third, apomictic plants are almost exclusively polyploids [10], which may be selected for the masking effects of deleterious mutations by unmutated chromosome copies [9, 23]. Though polyploids would supposedly have an increased absolute number of mutations because of a higher number of possible mutation sites, recessive mutations would not become expressed in the heterozygous state [9, 12].

We hypothesize that two factors effectively counteract mutation accumulation in polyploid asexual plants: (1) facultative sexuality and (2) selection on reduced, recombinant sexual gametophytes. Strikingly, a transcriptome study of facultative asexual hexaploid lineages of the *Ranunculus auricomus* complex revealed that dN/dS ratios did not significantly differ between sexual and asexual species. Asexuals showed no signs of genome-wide mutation accumulation despite ca. 80 kya of hexaploid genome evolution [24]. These hexaploid members of the *Ranunculus auricomus* species complex exhibit facultative gametophytic aposporous apomixis, where asexual development with unreduced gametophytes runs initially in parallel with the sexual development with reduced gametophytes [25] (shown in Fig. 1). Evidence of low to medium recombination rates was so far inferred only from natural populations [26]. Here we aimed to estimate (1) the recombination rate per generation using progeny arrays, (2) differential effects of purging selection on reduced/unreduced gametophytes by calculating abortion rates, and (3) the speed of mutation elimination in the hexaploid genome under the observed recombination rate and three selection scenarios via a mathematical model.

## Results

The three progeny arrays exhibited similar genotype diversities [*D*_(*V* − *progeny*)_ = 0.19, *D*_(*T* − *progeny*)_ = 0.20, *D*_(*I* − *progeny*)_ = 0.28] with 5–8 genotypes per progeny array. Pairwise genetic distances between genotypes showed that most progenies were clonal and identical to the respective mother genotype, but non-maternal offspring appeared in all three progenies. Character incompatibility methods revealed recombinants in the T and V clones. After averaging proportions of recombinant genotypes over the three progeny arrays, the mean number of recombinants as a proxy for recombination rate per generation was computed as *r* = 0.061 (Table 1).

**Table 1:** Genotyping using microsatellite analysis of three progenies of *R. carpaticola* × *R. cassubicifolius*. C = maternal clone, M = SSR mutant clone, R = recombinant

Progeny array (mother plant ID)	No. of progenies	No. of offspring genotypes	No. of C, M and R plants per progeny array	% of recombinant genotypes per progeny arrays
V (35/28)	39	8	31 (C), 4 (M), 4 (R)	10.26
T (29/15)	38	5	34 (C), 1 (M), 3 (R)	7.89
I (8492/27)	30	6	25 (C), 5 (M), 0 (R)	0.00
Mean	36	6	30 (C), 3 (M), 2 (R)	6.05

Starting at time *t* = 0 generations after a new deleterious mutation has become fixed in a non-recombining clone, the frequency of mutant clones *κ*_*m*_ = 1. A facultative asexual plant population with a non-zero recombination rate per generation and with one fixed deleterious mutation consists of three different offspring classes in the first generation (Additional file 3a). The frequencies of genotypes are given as: *ρ*_0_ (non-mutant recombinant from sexual seeds; ‘R’ genotype in Additional file 3a-b), *ρ*_*m*_ (mutant recombinant from sexual seeds; ‘R’ genotype marked with an asterisk in Additional file 3a-b), *κ*_*m*_ (mutant clone from apomictic seeds; ‘C’ genotype marked with an asterisk in Additional file 3a-b). We assume that recombination operates in all subsequent generations with the empirically estimated rate *r* = 0.061 or ~ 6% of recombinant genotypes per generation and that further generations can continue to produce both recombinant and clonal offspring (‘R’ and ‘C’ genotypes in Additional file 3a-b). Hence, in the second generation, the mutant clonal and mutant recombinant mothers each can produce three offspring classes (i.e., non-mutant and mutant recombinants and mutant clones). In contrast, the non-mutant recombinant mother can produce non-mutant clonal offspring (‘C’ in Additional file 3a-b) with a frequency given as *κ*_0_ in the second and following generations (Additional file 3c).

If selection *s* = 1.000, any mutated gametophyte is eliminated. Considering a selection *s* = 0.520 on female sexual gametophytes only (as approximated by the abortion rate), not only non-mutant recombinants (*ρ*_0_ = *r*|*s*–2|^− 1^) but also mutant recombinants (*ρ*_*m*_ = *ρ*_0_|1–*s*|) might produce offspring. Over generations, the frequency of mutant recombinants decreases with *ρ*_*m*_*(t)* = *ρ*_*m*_exp(*−ρ*_0_*t*). Similarly, the frequency of mutant clones *κ*_*m*_*(t)* = (1-*ρ*_*m*_)exp(*−ρ*_0_*t*) declines with time:

1 $$ {\kappa}_m(t)=\left(1-\frac{r\left|1-s\right|}{\left|s-2\right|}\right){e}^{-\frac{rt}{\left|s-2\right|}} $$

**Fig. 2:**
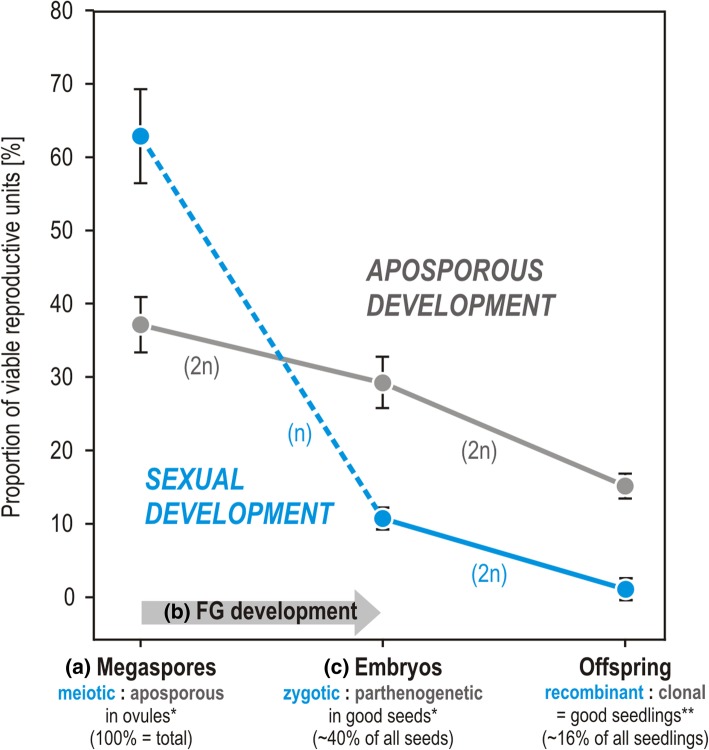
Abortion rates of viable reproductive units during sexual and aposporous development. Development starts from the megaspore phase (**a**), continues with gametophyte (**b**) and the embryo phase (**c**), and ends with the seedling phase. The major abortion (mean 52%) happens during the reduced female gametophyte (FG) phase (**b**) of the sexual development (blue dashed line). In comparison, the decline is less steep during the unreduced aposporous female gametophyte phase (solid gray line). These results suggest stronger selection upon the reduced phase (n) of the sexual development than upon the unreduced phase (2n) of the aposporous development. The decline from the embryo stage to the offspring (seedling) stage is in sexual and apomictic offspring almost the same, as germination rates are not significantly different. Proportions of reproductive units are given as means with confidence intervals. Data were adapted from [25, 26]

The decay steepness (Fig. 2) is more dependent on the rate of recombination than on the strength of selection because selection can act only on homozygous mutant recombinants (*ρ*_*m*_). Based on the Eq. (1) we revealed an estimate of time (*t*) needed to reach an arbitrary minimum frequency of mutant clones *κ*_*m,min*_, where mutants can be considered as eliminated or lost by genetic drift. Considering any value of *κ*_*m,min*_, the time necessary to reach it corresponds to *t (κ*_*m,min*_*)* = ln[(1-*ρ*_*m*_)*κ*_*m,min*_^− 1^]*ρ*_0_^− 1^, or expressed with recombination and selection:

2 $$ t\ \left({\kappa}_{m,\mathit{\min}}\right)=\ln \left[\left(1-\frac{r\left|1-s\right|}{\left|s-2\right|}\ \right){\kappa_{m,\mathit{\min}}}^{-1}\right]\frac{\left|s-2\right|}{r} $$

Any generation with non-zero recombination rate releases non-mutant clones with frequency *κ*_0_*(t)* = 1-[(*ρ*_0_ + *κ*_*m*_*(t)*)], or:

3 $$ {\kappa}_0(t)=1-\frac{r\ \exp \left(-\frac{rt}{\left|s-2\right|}\right)}{\left|s-2\right|} $$

Even if mutation accumulation operates rapidly in small populations, a constant recombination rate of 6% restores the least-loaded genotype class within a single generation cycle. According to the Eq. (2), the elimination of all mutant clones (e.g., *κ*_*m,min*_ < 0.001) takes approximately 138 generations for *s* = 1.000, 204 generations for *s* = 0.520 or 246 generations for *s* = 0.212 (Fig. 2). However, if obligate apomixis without any recombination is assumed, Muller’s ratchet would act rapidly in our model system. Depending on the model used, the ratchet would click between 32 and 3570 generations (Table 2).

**Table 2:** The speed of Muller’s ratchet under obligate asexuality, assuming fixed population size N = 10^3^ individuals and deleterious mutation rate U = 1.116. *n*_0_ = Number of individuals within the least-loaded class, s = selection coefficient, *t*_(J)_ = interclick time estimate based on Eq. 34 in [28], *t*_(NS)_ = interclick time estimate based on Eq. 33 in [29] and *t*_(ME)_ = interclick time estimate based on Eq. 24 [30]; all time estimates are given as numbers of generations per “click”. Bold text = estimates of the selection coefficients of the fastest ratchets; the (J)-value was computed according to Eq. 34 in [28], corresponding to a U-shaped function with a local minimum at *s* = 0.212, which we considered as a critical value of the fastest ratchet acting on sporophytes

*s*	*n*_0_	*t*_(J)_	*t*_(NS)_	*t*_(ME)_
0.340^(ME)^	38	6649	3008	**3570**
0.218^(NS)^	6	33	**32**	14,006
0.212^(J)^	5	**33**	32	16,146

## Discussion

We present here for the first time a model of mutation elimination in facultative asexual plants under consideration of empirical estimates of recombination rate, selection on gametophytes, and polyploid genomes in hexaploid *Ranunculus auricomus*. Our model provides support for the hypothesis that low rates of facultative sex prevent genome-wide mutation accumulation even in polyploids, as it was actually observed in a transcriptome study of the same model system [24].

### Recombination rates

**Fig. 3:**
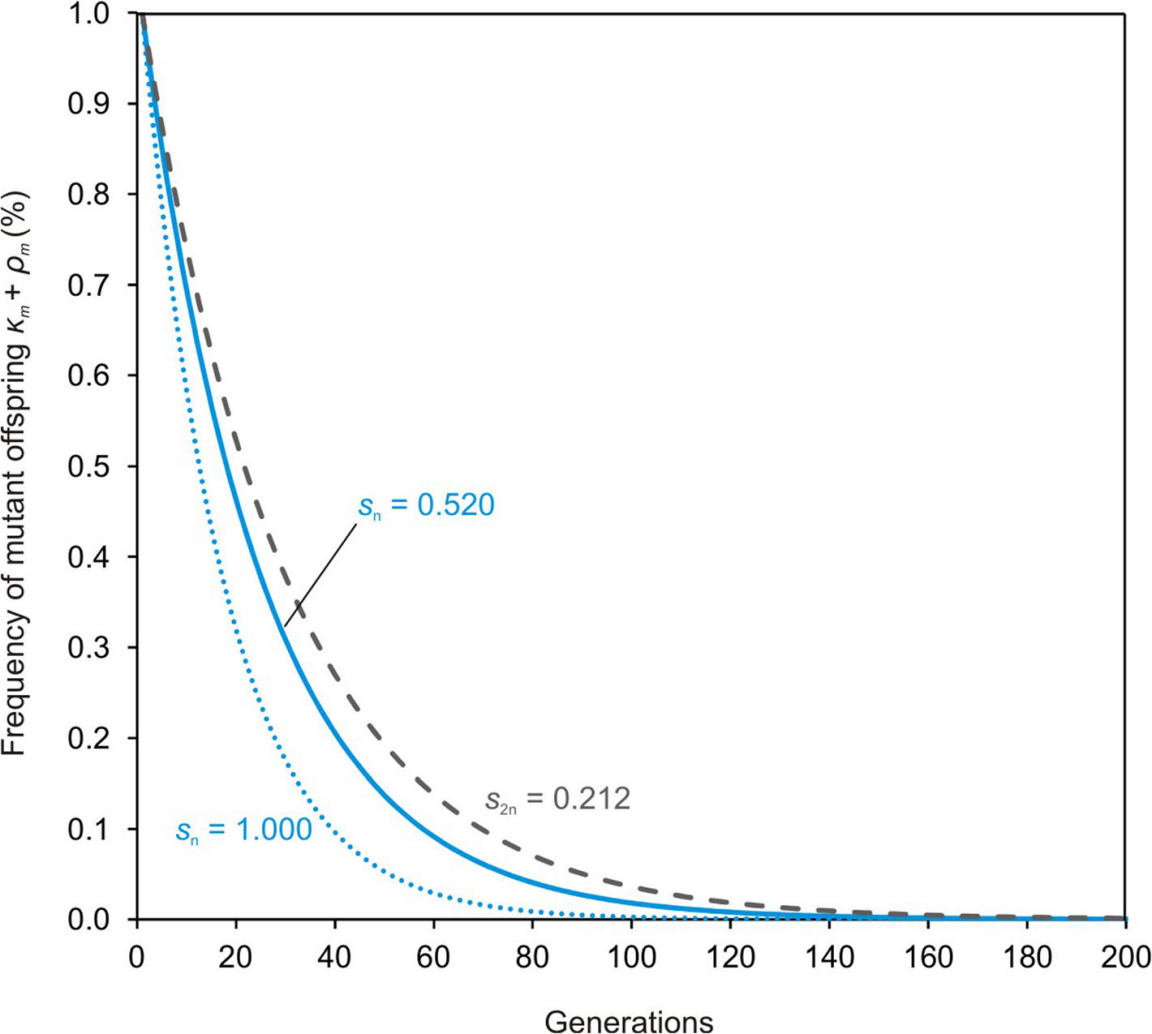
Model of mutant offspring decline under constant recombination rate per generation (*r* ≈ 6%) and three selection coefficients. The strongest selection (*s* = 1.000) on female and male gametophytes eliminates the mutation in ~ 138 generations (dotted blue line). The medium selection coefficient (*s* = 0.520), approximating selection on female gametophytes only, eliminates the mutation after ~ 204 generations. The weakest selection coefficient (*s* = 0.212; derived from [27]) represents selection on the sporophyte only; the mutation is eliminated after ~ 246 generations

We estimated recombination rates as a number of recombinants per sporophyte generation with microsatellites derived from the nuclear transcriptome dataset [24]. Directly after meiosis, the actual meiotic recombination rate is probably higher according to proportions of megaspores formed (Fig. 3). However, since female gametophytes are few-celled and deeply embedded in tissues, and because of the high chromosome numbers, it is not feasible to quantify meiotic recombination directly with cytogenetic methods. By calculating final recombinant offspring genotypes we rather use a proxy of a minimum recombination rate that actually occurs during the whole sexual development. Sorting out SSR variants is especially important for our study, as SSR loci can show a high mutational dynamics in hexaploids [31]. By using standard character incompatibility methods to discriminate SSR variants from recombinants [26, 32, 33], we avoid a critical overestimate of recombination rates. Overestimates would invalidate our model, while underestimates would just mean that the speed of mutation elimination could be even faster.

Recombination rate mostly depends on the degree of facultative sexuality, a factor which considerably varies among flowering plants. In general, plants with alternative meiotic and apomictic development in the same ovule, as it is the case in apospory (Fig. 3) or adventitious embryony, have higher degrees of facultative sexuality (i.e., higher proportions of sexually formed seed in one seed generation). These developmental pathways are found in almost 90% of all apomictic plant genera [34]. Hence, our study can serve as a model system for the great majority of apomictic plants. By using averaged data from different environmental conditions [35], we covered a broad range of naturally occurring variation in proportions of sexual versus apomictic seed formation. Indeed, natural populations of *R. carpaticola* × *R. cassubicifolius* exhibit a comparable genetic variation [26]. Some other apomictic plant genera show developmental pathways where meiosis is either restitutional or mitosis-like (e.g., diplospory [19]) or with suppressed recombination (e.g., permanent translocation heterozygosity; [36]). In these cases, alternative sexual pathways cannot be easily realized, because meiosis itself is altered, and there is no developmental phase where sexual and asexual gametophyte development could exist in parallel within one ovule. Hence, these forms of apomixis tend to be more obligate [19]. Indeed, transcriptomes of *Oenothera* (a rare case of obligate asexuality due to translocation heterozygosity; [36]) and genomes of diplosporous *Boechera* (except for conserved coding sites; [37]) showed signatures of mutation accumulation. These cases are in accordance with our results that maintenance of high levels of facultative sexuality, as in *Ranunculus*, and certain levels of recombination are crucial for the suppression of mutation accumulation in apomictic plants.

### Purging selection during reduced phases of the plant life cycle

Our results support the hypothesis that purifying selection at haplontic stages of the life cycle is an important benefit of sexuality [12]. For plants, the prolonged gametophyte phase with many genes expressed appears to be the main target of purifying selection. This has been shown for pollen [38] and hypothesized for female gametophytes based on previous developmental studies [39]. Here we demonstrate the same mechanism for female development in *R. carpaticola* × *R. cassubicifolius*. We used three selection coefficients to estimate mutation elimination via purging selection. The medium selection coefficient *s* = 0.520 was taken as an approximation to the fitness decline of ca. 52% during the female sexual pathway, inferred from the observed proportions of functional megaspores to sexually formed seeds (Fig. 3). However, regarding recombination and purging selection acting on male gametophytes as well (e.g., [40]) and additive effects after fusion of male and female gametes, the efficacy of purifying selection in the sexual pathway can be increased by various mechanisms. Self-pollination is frequent in apomictic plants, probably as a consequence of a breakdown of self-incompatibility systems in polyploids; selection acts for self-fertility especially in pseudogamous apomictic plants [41]. After self-fertilization of the egg cell with mutation carrying pollen, the mutation gets the double dosage in the zygote and consequently in the embryo, and hence will be exposed to selection during the sporophytic phase [11]. This happens not only after self-pollination within the same plant (i.e., within the same flower or between flowers of the same individual) but also after “cross-pollination” between clone-mates of a population, i.e., between genetically identical individuals. Furthermore, gene conversion during meiosis could make the mutation homozygous as well [11]. Gene conversion was considered an important mechanism preventing mutation accumulation in ancient asexual animals [42]. Although we have no data on these mechanisms, we regard a strong additive selection coefficient of *s* = 1.000 possible.

### Mutation rate estimates and Muller’s ratchet

There are multiple factors possibly acting against Muller’s ratchet, e.g. epistatic interactions, beneficial mutations, compensatory mutations and gene conversion. However, the purpose of our model is to illustrate the effect of deleterious mutations. We show that even in the absence of all the above mentioned corrective factors, a bit of recombination suffices to prevent facultative asexuals from mutational meltdown. Concerning the beneficial mutation rates, there are two lines of evidence, the theoretical and experimental. Theoretical models suggest that epistatic effects can effectively halt Muller’s ratchet [28] or that already a low amount of beneficial mutations might have the same effect [43]. However, in the fast ratchet regime, only a considerable amount of beneficial mutations might prevent an asexual population from extinction [43]. The authors confirmed that a combined effect of beneficial mutations and facultative recombination has not been explored even at the theoretical level, and synthesis of a common model including deleterious and beneficial mutations is missing. A full analysis of this complex situation is clearly beyond the scope of our study. Transcriptome data from *R. auricomus* revealed only a small amount of outlier genes under positive selection [24], and hence we assume effects of potentially beneficial mutations as negligible. Another line of evidence, the experiments on model organisms, shows considerable discrepancies not only among studied organisms (yeasts, viruses, *Arabidopsis*, *Daphnia*) but also among experimental approaches showing differences between laboratory and field conditions [44]. Some studies suggest negligible rates of beneficial mutations [45, 46], others postulate that a considerable proportion of genomic mutations is beneficial [47, 48]. Observations of extremely high beneficial mutations rates are considered to be experimental artifacts [44]. The possible explanation for the surprisingly high beneficial mutation rates observed by some authors [44] might reflect specific environmental conditions or experimental setup or specific fitness of founder genotypes [44]. An unexpectedly high ratio of beneficial: deleterious mutations might also result from a low detectability of phenotypic variation due to slightly deleterious mutations [49].

Our model suggests that facultative sexuality in polyploid *Ranunculus auricomus* halts Muller’s ratchet, because (1) the least-loaded class is restored in every generation cycle, and (2) mutant frequency decreases with persistent low recombination rates and strength of selection on gametophytes. However, in the obligatory absence of sex, Muller’s ratchet might operate quickly, particularly in very small populations [50, 51]. Considering a small effective population size of hexaploid *R. carpaticola* × *R. cassubicifolius* and a relatively high deleterious mutation rate adapted from [52], we estimated the critical selection coefficient, which is responsible for the fastest ratchet ‘clicks’. This critical value of *s* approaches 0.212, 0.218 or 0.340, depending on whether computed according to [28, 29], or [30], respectively. Under these selection scenarios without haplontic phases, and without any recombination, the ratchet could click in the worst case scenario within 32 generations (Table 2).

However, this scenario is unrealistic as facultative sexuality is in plants a persistent mechanism. The genetic and epigenetic control mechanisms of apomixis mostly represent just ectopic or asynchronous mis-expressions of the same genes regulating the sexual pathway [53]. The (epi) genetic control factors of apomixis remain in the heterozygous or hemizygous state with the wild-type (epi) alleles [53, 54]. The apomixis-controlling loci represent large, non-recombinant genomic regions and are inherited as dominant factors [54]. Therefore, apomixis-controlling alleles or epialleles cannot become homozygous. Since expression of apomixis is dependent on allele dosage [55], apomixis can never become obligate. Apomixis is just superimposed on the sexual pathway [53]. The option of an alternative sexual reproductive pathway, or even the return to obligate sexuality, is present. Interestingly, plant species with a lower degree of asexuality and signatures of mutation accumulation are diploids [36]. The lower mutation rate of diploid genomes might help these lineages to survive with “almost no sex” for some time. For polyploid apomicts – the typical condition for most apomictic plants - maintenance of low levels of facultative sexuality prevents mutation accumulation. Hence, long-term evolution of facultative apomictic plant lineages is probably not limited by the threat of Muller’s ratchet despite polyploidy.

## Conclusions

We tested here for mutation accumulation in a typical apomictic plant model system with a big polyploid hybrid genome, but still exhibiting facultative sexuality in both female and male development. We used here the novel approach of combining empirical data and mathematical modelling. Our results confirmed that even in a hexaploid genome, the empirically observed low frequencies of facultative sexuality (c. 6% of recombinants per generation) suffice to prevent accumulation of deleterious mutations via Muller’s ratchet. The efficiency of purging selection is in the sexual pathway of flowering plants strongly increased by acting on the haplontic (meiotically reduced) gametophyte stage, in which many genes are expressed. Hence, previously masked recessive mutations are exposed to purging selection in the gametophyte. Results provide a general explanation for maintenance of a little bit of sex in polyploid asexual flowering plants.

## Methods

Our model is based on empirical estimates of recombination rate, selection coefficients, and genomic mutation rate for the hexaploid ancient hybrid *Ranunculus carpaticola* × *Ranunculus cassubicifolius,* a member of the Eurasian *R. auricomus* complex [24, 27, 35]. Voucher specimens were deposited in the herbarium of the University of Goettingen (GOET).

### Recombination rate per generation

As a proxy for recombination rates, we determined the mean proportion of recombinant genotypes per offspring generation by using microsatellite markers. This approach is most feasible when phenotypic markers for studying segregation are not available [56]. Cytogenetic meiosis studies for assessment of recombination rates are so far not possible in our model system because of the difficulties to observe female plant meiosis directly, and the high chromosome numbers (2*n* = 48). To estimate a minimum recombination rate, we produced progeny arrays of three allohexaploid mother plants from geographically separated populations belonging to the ancient hybrid *Ranunculus carpaticola* × *Ranunculus cassubicifolius* [24, 27, 35].

*Ranunculus auricomus* is a long-lived plant with a long generation turnover (2–3 years) and hence we could analyze just one generation. We selfed the three mother plants under controlled garden conditions, following the rationale of [41], and generated 30, 38 and 39 F1-offspring per mother plant (“I”, “T” and “V”, respectively; Table 1). We genotyped 107 plants with six nuclear microsatellite loci previously developed out of the RNA-Seq data of [24]. We used the five SSR loci and protocols from [35] and one additional locus R2252 (forward primer: TCGGGTTCACCCACTAAATC, annealing temperature = 60 °C; reverse primer CATGGACTAGTTTCCGCCAT, annealing temperature = 60 °C). The plants are hexaploid with up to six alleles per single locus, and allele copy number in a genotype cannot be reliably scored from electropherograms. Hence, we scored the fragments as dominant and recorded presences and absences of each allele across all analyzed individuals in a binary matrix (Additional file 1). In the next step, we compared the F1-offspring multilocus genotypes with their respective mother plant genotypes and estimated the total number of genotypes and genotype diversity D (Nei’s corrected genetic diversity) in the software GENOTYPE/GENODIVE [57]. In the same program, we computed a matrix of pairwise genetic distances between all genotypes assuming the infinite allele model (IAM; [58] and plotted them as histograms (Additional file 2).

Since the recombination rate has the strongest effect on mutation elimination, overestimates of recombinants per generation would invalidate the model. In SSR studies, this could happen if non-maternal SSR variants that have just arisen from replication slippage [59] would be scored as recombinants. In fact, hexaploid plants can show a high mutational dynamics of SSR variants [31]. To discriminate true recombinants from non-maternal SSR variants we used three character incompatibility methods. We first used character incompatibility analysis as implemented in the module Jactax of the program PICA 4.0 [60]. Any two characters (alleles of the binary matrix) are incompatible with a hierarchical, tree-like divergence pattern if they show all four character state possibilities in at least four different genotypes A, B, C, D (e.g., A: 1/0, B: 1/1, C: 0/1, D: 0/0) which is a strong signal for recombination [61]. Single novel character states or combinations that are compatible with a hierarchical pattern (e.g., A: 1/0, B: 1/0, C: 0/1, D: 0/1) are a signal for mutations in the marker system. The requirement of at least four different genotypes and two different loci was met in all three progeny arrays (Additional file 2). Character compatibility analysis calculates an initial matrix incompatibility (MI), and subsequent stepwise removal of recombinant genotypes contributing to MI finally identify mutant genotypes with MI = 0 (Additional file 2). Second, the same F1-genotypes were concordantly depicted by a recombination network analysis and visualized within a recombination cycle using the software SplitsTree 4.0 [62]. Here, distance-based incompatible splits are visualized as rectangles, in which recombinants are placed at nodes while mutant genotypes appear as terminal branch-offs (Additional file 2: Figure S2). This network topology was further confirmed with a NeighborNet analysis [62] for which a statistical test (1000 bootstrap replicates) was conducted (Additional file 2). Finally, we considered genotypes as recombinants only if they were determined by all three character incompatibility methods. We averaged the observed proportions of recombinant F1-offspring within each of the three progeny arrays (i.e., recombination rate per generation, *r*).

### Selection coefficients

Selection on gametophytes during development is an essential part of our plant-specific model [11]. Previous histological observation of gametophyte development on different apomictic plant species showed various forms and stages of abortive phenotypes and indicated different proportions of sexual vs. apomictic gametophytes during developmental stages [25, 39, 63]. Sexual and apomictic gametophytes develop concurrently in adult plants, they do not differ morphologically, but they have allele dosage-dependent gene expression levels [64]. Hence, we assume that abortion rates of reduced sexual vs. apomictic unreduced gametophytes are a result of the different strength of selection on expressed versus masked deleterious mutations. We re-analyzed published developmental data for the *Ranunculus carpaticola × Ranunculus cassubicifolius* progenies [35, 65] to estimate the abortion rates during the female gametophyte phase as an indirect indicator of the strength of selection. First, for the stage directly after meiosis, we averaged the proportions of unreduced aposporous initial cells (AIC; see Fig. 1) and reduced functional meiotic megaspores from microscopic observations of ovules (data from [35]). These data averaged the observed variation under both stressed and unstressed conditions, as they may occur in nature. Second, apomictic versus sexual pathways of seed formation were determined using flow cytometric seed screening, and the averaged proportion of well-developed sexual versus asexual seeds was calculated from mature fruits (data from [35]). Third, averaged seed germination rates were derived from [25] and, finally, we assessed the proportion of viable recombinant and clonal offspring based on our estimate of the average recombination rate (see Table 1). Proportions of sexual vs. apomictic development were calculated from proportions of surviving initial cells/seeds/progenies at each developmental step (as only these can continue development) to reveal the actual fitness differences of sexual and apomictic development during the gametophytic and the sporophytic phases (see Fig. 1).

We compared proportions of abortions between different developmental phases, i.e., megaspores, embryos and offspring/seedlings (Fig. 3). As expected, we observed higher abortion rates in the reduced (sexual) than in the unreduced (aposporous apomictic) female gametophyte (Fig. 3). The observed mean abortion rate of ca. 52% in reduced/sexual female gametophytes (Fig. 3), was taken as the argument for a medium selection coefficient (*s* = 0.520).

However, purifying selection is possible in male, reduced gametophytes as well [38]. Not only female gametophytes are partly aborted in apomictic *R. auricomus* but also pollen [25, 28, 52], where selection against deleterious mutations acts on the male gametophyte as well. Hence, we also take a proxy of *s* = c. 0.5 for male development. Since sexual development involves the fusion of male and female gametes that have been both purged from mutations, effects of selection will be additive. This justifies also for testing a strong selection coefficient (*s* = 1.000). For an estimate of selection on adult plants (i.e., sporophytes only), we computed a theoretical model based on mutation fixation (with estimated finite population size) following [28], which is outlined below.

### Modeling of mutation elimination for polyploids

Deleterious mutation rates in plants are *U* ≈ 0.1–2.0 per genome and generation (for mutations with mean selection coefficients in the range *s* ≈ 0.1–0.2) [1]. To estimate deleterious mutation rate in hexaploid *Ranunculus auricomus*, we multiplied the estimate for diploid *Arabidopsis U* ≈ 0.1 [1, 52, 66] with the correction factor of 11.16 by which the hexaploid *Ranunculus* genome size is bigger (*G*_6*x*_ = 15.69 pg; 2C) [26]. By comparison, the genome of another small-statured plant *Amsinckia spectabilis* is four times as large as an average *Arabidopsis* genome [67] and it exhibits approximately four times higher *U* than *Arabidopsis* [68]. The correction factor of 11.16 we use for *Ranunculus* already regards a diploid versus a polyploid chromosome complement. We prefer the correction factor based on absolute genome size rather than on ploidy level, because *Ranunculus auricomus* shows ‘genome downsizing’ in polyploids [69], i.e., the increase of hexaploid genome size is lower than expected by multiplication of haploid genome size with ploidy level. Genome downsizing is a frequent phenomenon in polyploid flowering plants [70]. A more detailed estimate of polyploid genome evolution is not yet feasible as no completely sequenced reference genome is available for *Ranunculus*. We did not regard in our model rare beneficial mutations which can also halt Muller’s ratchet [43] because the *Ranunculus* transcriptome dataset [24] included only a small amount of outliers of non-synonymous mutations. Since even these mutations are not necessarily beneficial [24], we regard the effect of beneficial mutations as negligible. We further did not consider negative epistatic interactions of mutations, which may theoretically increase effects of deleterious mutations, but are relatively uncommon [71].

We assume a mutation (A) with dominance at 1/3 allele dose (i.e., expressed as Aaa in sexual triploid gametophytes but not as Aaaaaa in hexaploid asexual gametophytes or in sporophytes) due to dosage effects. We assume for sexual development predominant bivalent formation at meiosis, regular segregation, and disomic inheritance, as is typical for allopolyploids in which doubled homologs can pair [72] because *Ranunculus carpaticola* × *Ranunculus cassubicifolius* is an allopolyploid hybrid [24, 73]. We further assume non-overlapping generations and that mutation-carrying and mutation-free offspring are released in constant proportions every generation, resulting in an exponential decay of mutant frequency with time (Additional file 3). We assume a low but constant average recombination rate, exposing mutation-carrying gametes to purging selection. Based on these parameters, we investigated an influence of three selection coefficients.

### Mutation fixation

We estimated the time needed for a slightly deleterious mutation to become fixed in a finite, obligate asexual population from the approximation of Muller’s ratchet inferred by [28] and compared to other approaches [29, 30]. We consider a small effective population size *N* = 10^3^ estimated from population genetic studies on natural 6x *R. carpaticola* × *R. cassubicifolius* which revealed a high F*st* value (0.82) [26], and from field observations of population size by the authors. The diploid mutation rate *U*_2*x*_ = 0.100 [52] was adapted for hexaploid *Ranunculus* (i.e., *U*_6*x*_ = 1.116) using the correction factor of 11.16, based on the ratio between an averaged DNA content of non-replicated holoploid genomes of small-statured model plants (e.g., *A. thaliana*) and hexaploid *R. auricomus* genotypes [26].

We calculated a theoretical selection coefficient with presumably highest effect on the “mutational meltdown” of the population [13, 50, 74], inferred from the critical range of selection coefficients which favor the fastest ‘clicking’ of Muller’s ratchet. The critical selection coefficient was computed with the parameter set above as *s* = 0.212. We consider it as an approximation of selection against deleterious mutations in hexaploid sporophytes only. We regarded the parameter regime of *λ* = *U*/*s* (=1.116/0.212) ≈ 5.3 as the ‘worst scenario’, where Muller’s ratchet clicks very frequently in a small obligatory asexual population (*N* = 10^3^ plant individuals). We inferred the ratchet speed based on several published approximations (Table 2).

## Additional files

Additional file 1: Microsatellite data matrix. (XLSX 28 kb)

Additional file 2: Graphical representation of results of the three character incompatibility methods for identification of recombinants. The graphs visualize results of calculations. (DOCX 249 kb)

Additional file 3: Model of mutation elimination under constant recombination rate and selection on female gametophytes. The graph visualizes the model for the first generations. (DOCX 264 kb)

## Data Availability

All data generated or analyzed during this study are included in this published article [and its supplementary information files].

## Abbreviations

dN/dS: The ratio of substitution rates at non-synonymous and synonymous sites
F1: The first filial generation of offspring of different parental species
Fst: The fixation index
kya: Thousand years ago
RNA-Seq: Whole transcriptome shotgun sequencing
SSR: Simple sequence repeats
