# Supplementary material for: A little bit of sex prevents mutation accumulation even in apomictic polyploid plants
Source: BMC Evol Biol. 2019 Aug 14;19:170. doi: 10.1186/s12862-019-1495-z (PMC6694583; doi:10.1186/s12862-019-1495-z)
Supplement: Supplementary file 2 — Graphical representation of results of the three character incompatibility methods for identification of recombinants. The graphs visualize results of calculations. (DOCX 249 kb) [file 12862_2019_1495_MOESM2_ESM.docx]

**Additional file 2. Graphical representation of results of the three character incompatibility methods for identification of recombinants.**


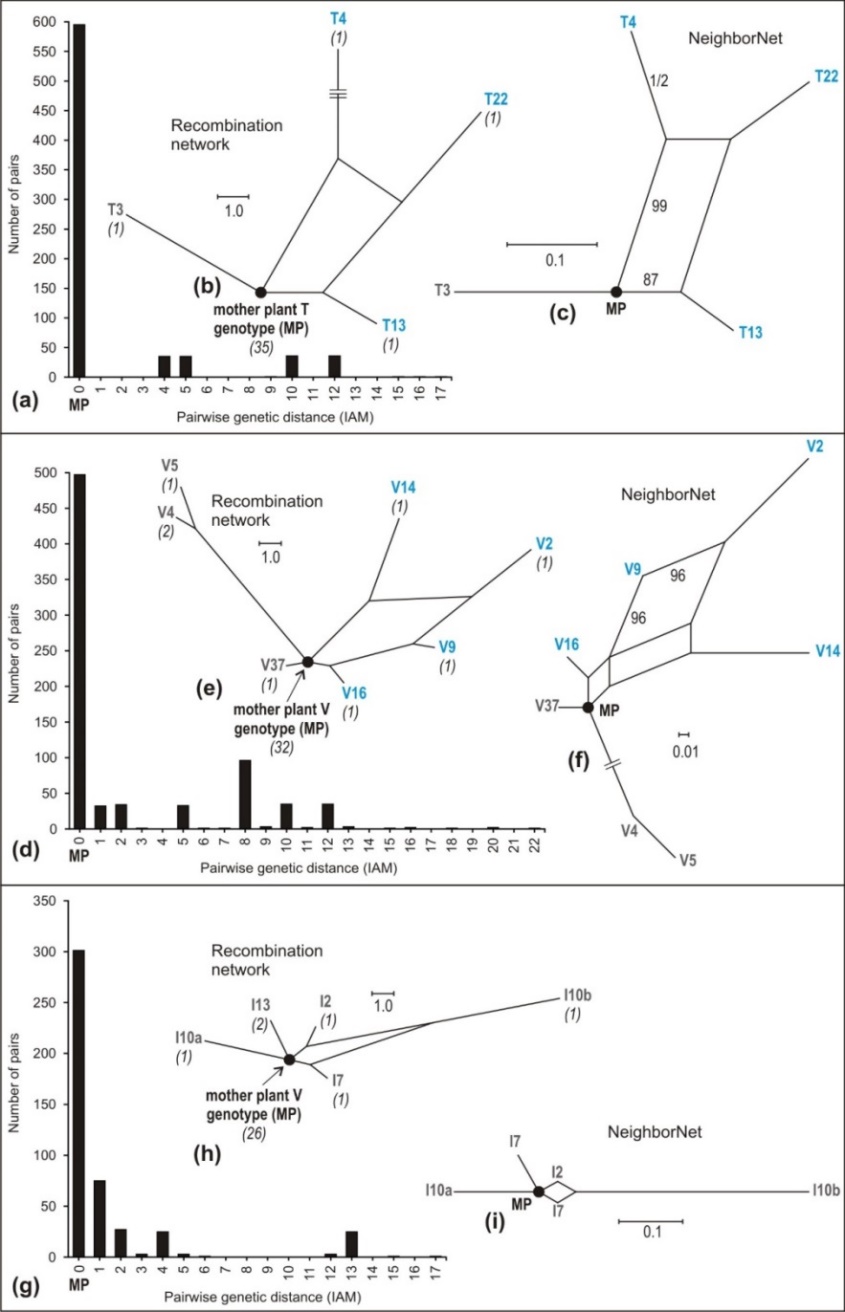


**Additional file 2 Figure.** Distinguishing clonal versus recombinant genetic variation (SSR data) in progeny arrays. (a)-(c) T-progeny analyses, (d)-(f) V-progeny analyses, (g)-(i) I-progeny analyses. (a), (d), (g) Histograms of pairwise distances among genotypes under infinite allele mutation model (IAM). Very low genetic distances close to 0 indicate clonality and higher genetic distances indicate the presence of recombinant genotypes. (b), (e), (h) Recombination networks of the SSR data show mother plants (MP) genotype and its clonal (gray letters) and recombinant (blue letters) F1-progenies. Numbers in brackets indicate numbers of genetically identical progenies. Recombinant genotypes were detected in (b) and (e) as they participate on recombination cycles within the recombination networks. The blue highlighted individuals were congruently depicted as recombinants also in character incompatibility analysis in the software PICA. (c), (f), (i) NeighborNet analyses of the SSR data confirm the splitting structure seen in the recombination networks. The highest bootstrap values are given.

**
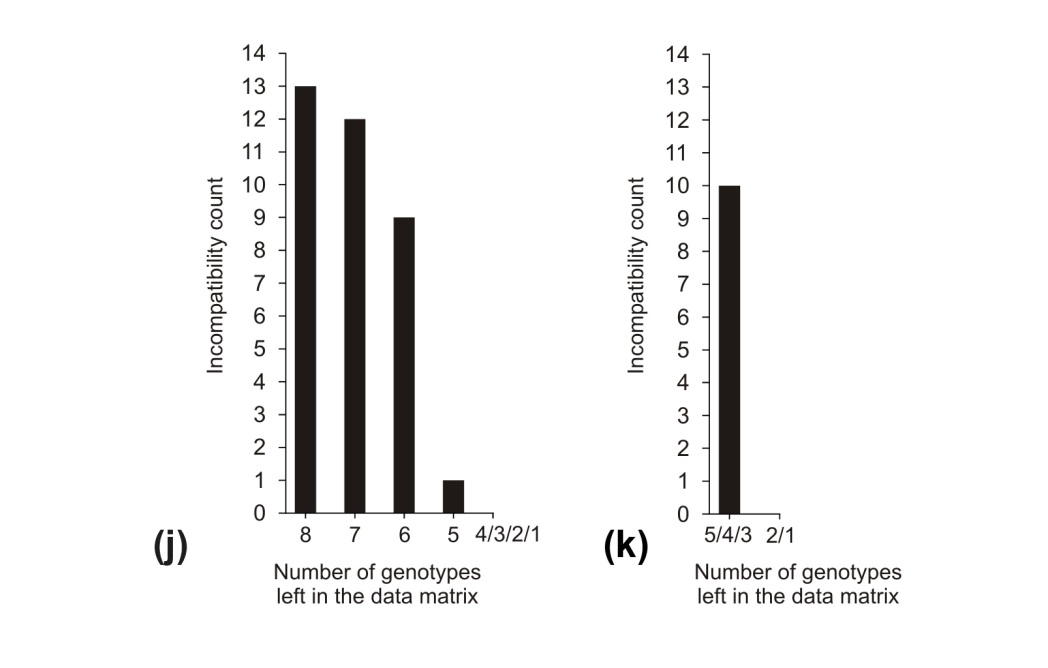
**

**continued.** Graph of character incompatibility of SSR multilocus genotypes, discriminating recombinant and non-recombinant genotypes (j) V-progeny array, (k) T-progeny array. The I-progeny array revealed no recombinants. Dark bars show reduction of matrix incompatibility (MI) upon successive deletion of recombinant genotypes contributing to matrix incompatibility. The number of genotypes left in the matrix represents non-recombinant genotypes (SSR variants = M).
