# Supplementary material for: A little bit of sex prevents mutation accumulation even in apomictic polyploid plants
Source: BMC Evol Biol. 2019 Aug 14;19:170. doi: 10.1186/s12862-019-1495-z (PMC6694583; doi:10.1186/s12862-019-1495-z)
Supplement: Supplementary file 3 — Model of mutation elimination under constant recombination rate and selection on female gametophytes. The graph visualizes the model for the first generations. (DOCX 264 kb) [file 12862_2019_1495_MOESM3_ESM.docx]

**Additional file 3. Model of mutation elimination under constant recombination rate and selection on female gametophytes.**


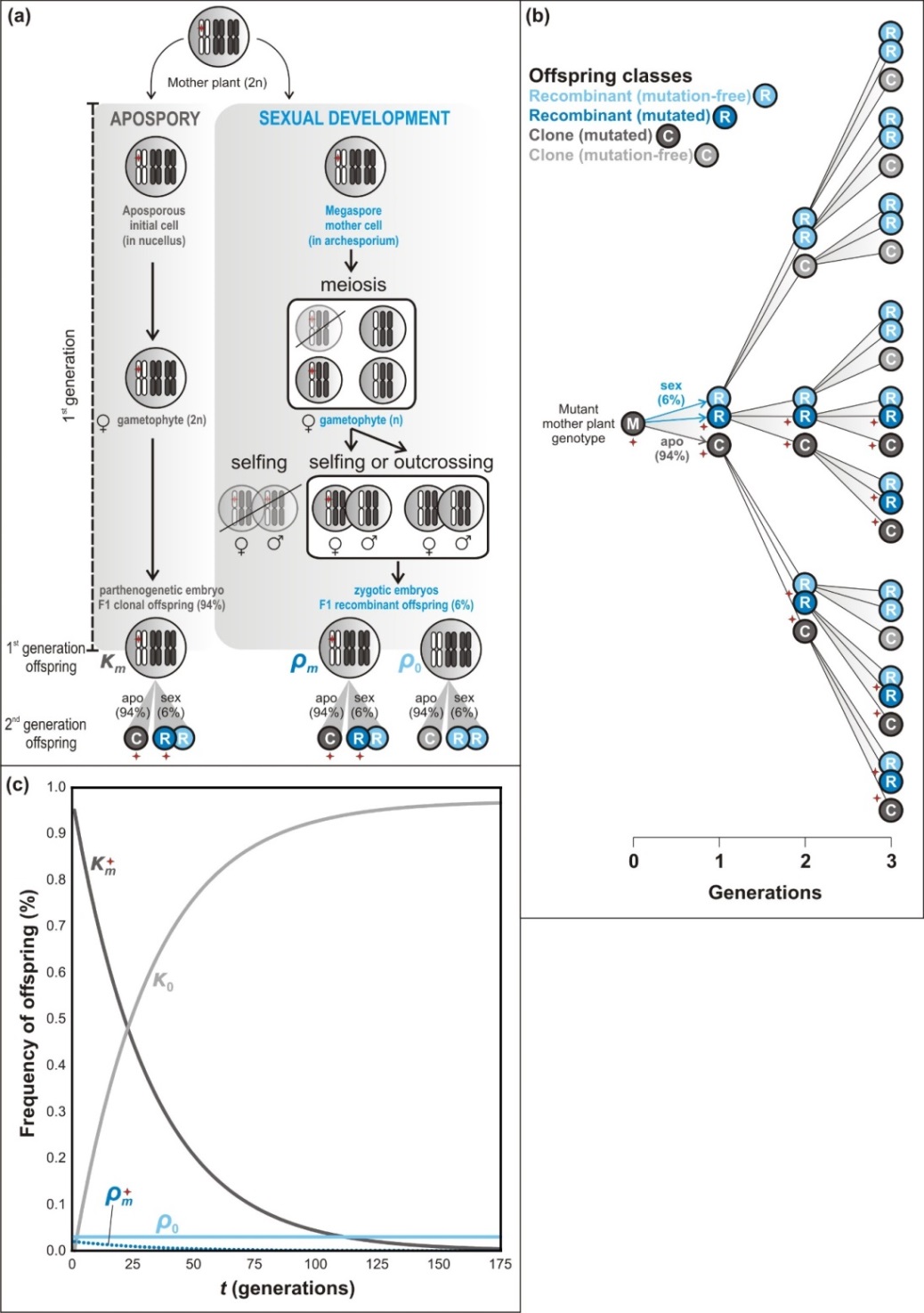


**Additional file 3 Figure. Model of mutation elimination under constant recombination rate and selection on female gametophytes.** (a) Aposporous and sexual development results in clonal and recombinant offspring, respectively. Grey circles with white “C” letter indicate clonal offspring; blue letters with white “R” letter indicate recombinant offspring; red asterisk stands for a mutation. The frequency of each offspring is given by *κ_m_* (mutant clone), *κ*_0_ (non-mutant clone), *ρ_m_* (mutant recombinant) and *ρ*_0_ (non-mutant recombinant). (b) The shift in frequencies of mutant and non-mutant offspring over the first three generations. (c) The shift in frequencies of mutant and non-mutant offspring modeled over multiple generations until mutants are eliminated.
